# A qualitative analysis to optimize a telemonitoring intervention for heart failure patients from disparity communities

**DOI:** 10.1186/s12911-016-0300-9

**Published:** 2016-06-24

**Authors:** R. Pekmezaris, R. M. Schwartz, T. N. Taylor, P. DiMarzio, C. N. Nouryan, L. Murray, G. McKenzie, D. Ahern, S. Castillo, K. Pecinka, L. Bauer, T. Orona, A.N. Makaryus

**Affiliations:** Department of Medicine, Hofstra Northwell School of Medicine, Manhasset, NY USA; Department of Occupational Medicine, Epidemiology, and Prevention, Hofstra Northwell School of Medicine, 175 Community Drive, Great Neck, NY 11021 USA; SUNY Downstate School of Medicine, 450 Clarkson Ave, Brooklyn, NY 11203 USA; Community Advisory Board, Northwell Health, 175 Community Drive, Great Neck, NY 11021 USA; Nassau University Medical Center, 2201 Hempstead Tpke, East Meadow, NY 11554 USA; Northwell Health, 175 Community Drive, Great Neck, NY 11021 USA; Department of Cardiology, Hofstra Northwell School of Medicine, Manhasset, NY USA

## Abstract

**Background:**

The use of telemonitoring is a promising approach to optimizing outcomes in the treatment of heart failure (HF) for patients living in the community. HF telemonitoring interventions, however, have not been tested for use with individuals residing in disparity communities.

**Methods:**

The current study describes the results of a community based participatory research approach to adapting a telemonitoring HF intervention so that it is acceptable and feasible for use with a lower-income, Black and Hispanic patient population. The study uses the ADAPT-ITT framework to engage key community stakeholders in the process of adapting the intervention in the context of two consecutive focus groups. In addition, data from a third focus group involving HF telemonitoring patient participants was also conducted. All three focus group discussions were audio recorded and professionally transcribed and lasted approximately two hours each. Structural coding was used to mark responses to topical questions in the interview guide.

**Results:**

This is the first study to describe the formative process of a community-based participatory research study aimed at optimizing telehealth utilization among African-American and Latino patients from disparity communities. Two major themes emerged from qualitative analyses of the focus group data. The first theme that arose involved suggested changes to the equipment that would maximize usability. Subthemes identified included issues that reflect the patient populations targeted, such as Spanish translation, font size and medical jargon. The second theme that arose involved suggested changes to the RCT study structure in order to maximize participant engagement. Subthemes also identified issues that reflect concerns of the targeted patient populations, such as the provision of reassurances regarding identity protection to undocumented patients in implementing an intervention that utilizes a camera, and that their involvement in telehealth monitoring would not replace their clinic care, which for many disparity patients is their only connection to medical care.

**Conclusions:**

The adaptation, based on the analysis of the data from the three focus groups, resulted in an intervention that is acceptable and feasible for HF patients residing in disparity communities.

**Trial registration:**

NCT02196922; ClinicalTrials.gov (US National Institutes of Health).

**Electronic supplementary material:**

The online version of this article (doi:10.1186/s12911-016-0300-9) contains supplementary material, which is available to authorized users.

## Background

Heart failure (HF) is a major contributor to differences in morbidity and mortality between racial, ethnic and income groups [1–5]. The relative incidence of HF is 50 % higher in Blacks, which occurs at an earlier age, resulting in more advanced disease severity and earlier death than Whites [6–8]. Similarly, Hispanics with HF are diagnosed younger and die earlier than non-Hispanic Whites. Reasons for this greater disease burden in underserved populations are complex, resulting from the interaction of factors such as comorbidities, health access, socioeconomics and cultural factors [7, 9, 10]. The disease burden of HF in these populations warrants a tailored, patient-centered management approach.

Telemonitoring (TM) is a promising approach to optimize outcomes for HF patients living at home [11]. As exacerbations are common, monitoring physiologic indicators, such as weight and blood pressure, facilitates improved management through timely treatment adjustments. Without leaving the home, vital signs can be transmitted and out-of-range values can be quickly addressed by clinicians. Using the audio/video component, TM involves the patient in self-monitoring and clinician feedback, allowing patients to stay in their homes while remaining connected to their support systems [12–14]. One meta-analysis of 14 randomized trials found that remote monitoring of HF patients reduced admission rates by 21 % and all-cause mortality by 20 %, while improving quality of life (QoL) [11]. Riegel‘s group reported higher patient satisfaction among telemonitoring patients, compared with usual care [15].

Although many TM studies have been published, there is limited literature on the use of TM in underserved populations. The few TM studies conducted in disparity populations have focused primarily on diabetes and blood pressure management. For example, the IDEATel study found that a TM intervention in older, medically underserved and ethnically diverse patients led to a net improvement in patients’ level of cholesterol, Hemoglobin A1c and blood pressure [16]. Another study found that African-American and Hispanic participants were less adherent than White participants to the diabetes intervention [17]. One hypertension study showed that TM was effective at decreasing blood pressure in Blacks [18]. A telephone-based case management intervention implemented in Mexican HF patients living in the U.S. failed to show significant differences in HF hospitalizations, cost of care, mortality and depression [19]. In a series of focus groups, George et al. reported that African-American and Hispanic participants were satisfied with TM’s ability to facilitate immediate access, but Hispanics expressed confidentiality concerns.

Studies suggest that developing culturally tailored interventions may improve patient satisfaction, program adherence and ultimately, clinical outcomes [20]. Given the discrepancy in HF disease burden between racial, ethnic and income groups, researchers must tailor effective interventions for acceptability and relevance for those in populations at greatest risk.

Community-based participatory research (CBPR) is a collaborative approach of community engagement in which researchers work in partnership with a variety of stakeholders with differing perspectives (including patients), to address the gap between science and “real world” practice through joint decision-making. These decisions may include: defining the research question, collection/analysis of data, interpretation of findings, and dissemination of results [21].

The qualitative process reported herein describes the adaptation a home TM program using a CBPR approach, in a population of Black and Hispanic HF patients from disparity communities in the New York Metropolitan Area, with the goal of optimizing program usability. This paper represents the formative phase of a mixed methods study that includes a randomized clinical trial (RCT) designed to assess whether telemonitoring is effective in improving care for African-American and Latino HF patients from disparity communities. The RCT recruits African-American and Latino disparity patients hospitalized with a primary diagnosis of heart failure to receive either the optimimized telemonitoring program in their homes or standard care (including home care and heart failure clinic-based care). The purpose of this first formative phase was to provide community and key stakeholder feedback to optimize the intervention for this target population. CBPR principles involve recognizing community members as “equal influencers” over the conduct of research, providing a replicable framework when adapting an intervention so that it is acceptable and effective in target communities [21, 22]. Using CBPR in the formative phase of a project not only helps to facilitate effective adaptation, but to ensure overall project effectiveness, as an intervention that is not acceptable in a particular population will be unlikely to be successfully replicated [23].

CBPR has been utilized in adapting/developing programs addressing the needs of disparity communities in a variety of areas including mental health [24], cancer [25], sexually transmitted infections [26–28] and smoking [29]. However, there is a dearth of literature regarding telemonitoring adaptation in HF patients from underserved communities. The current formative phase adaptation relied on features of the ADAPT-ITT [27] model, developed for adapting evidence-based HIV interventions. The ADAPT-ITT model involves stakeholders across multiple phases: 1) providing input into a needs assessment, 2) decision-making regarding program choice, 3) administering the intervention with theater testers, 4) producing a draft of the proposed intervention, 5) including topical experts in adaptation, 6) integrating all of the input into the new intervention, 7) training staff on revised intervention and 8) conducting a full intervention pilot test [27]. As described below, the current project involved two Community Advisory Board (CAB) focus groups, including theatre testing, followed by a patient participant focus group which served as a project pilot (see Table 1). The first CAB focus group addressed adaptation phases 1 and 5; the second CAB focus group addressed adaptation phases 3–7; and the final patient focus group addressed phase 8. Phase 2 was only partially implemented, as certain elements of the telemonitoring intervention were already selected based on previous research and grant award assurances.

**Table 1:** Community advisory board

Community advisory board members				
Patients and caregivers	Patient advocates	Health Care Practitioners	Health Policy and Finance	Disparities Expert
HF patient	Retired Deputy Commissioner of Health and Patient Advocate	Geriatrics and Chronic Care Management Expert	Policy Expert	Health Disparities Expert
Caregiver*	Health Access Specialist	Heart Failure Expert	Payor Expert	Social Disparities Expert*
HF patient	Telehealth Installation and Patient Orientation Specialist	Telehealth Nurse	Health Law Expert	
HF patient	Hispanic Community Leader	Pharmacist		
HF patient				
Caregiver				

*note: the asterisks indicate that this is referring to the same participant

## Methods

The data presented herein were collected during three focus groups. The first two were attended by the TM CAB members, comprised of key stakeholders, including: Black and Hispanic HF patients and nonprofessional caregivers; disparity experts, clinicians (geriatrician, HF expert, and a TM nurse), patient advocates, payor and health policy representatives (Table 2). The role of the CAB was to advise the study team on all aspects of study design, implementation, evaluation and dissemination. More specifically, the CAB was responsible for program tailoring, for identifying factors impacting acceptance/feasibility among this population to reduce the impact of such factors on usability. The primary goal of the first CAB focus group was to obtain specific feedback regarding intervention adaptation needs. The goals of the second CAB focus group were to theater test with HF patient stakeholder CAB members and ensure that the adaptation was successfully implemented. Although feedback from all CAB members was incorporated, the study team gave particular weight to patient stakeholder feedback. The third focus group involved TM patients who were randomized to the telemonitoring study arm and completed a three month pilot to identify ”on the ground” barriers.

**Table 2:** ADAPT-ITT framework [27]

ADAPT-ITT phase	Methodology
1. Assessment	Conducted focus groups/needs assessment with Community Advisory Board (CAB)
2. Decision	Decision regarding type of intervention was pre-determined by evidence base; however, decisions regarding characteristics of the intervention (equipment and study structure) were made.
3. Administration	Theater testing was conducted during the CAB focus groups with patient stakeholders prior to intervention implementation.
4. Production	A draft of the tailored intervention was presented to the CAB for further feedback and approval.
5. Topical experts	The study team specifically recruited topical experts for CAB membership; see Table 2.
6. Integration	CAB input was integrated into the final adapted version of the intervention; the telehealth software company revised the software to reflect CAB recommendations.
7. Training	Both clinical and installation experts received training with regard to study structure and equipment use.
8. Testing	A pilot test of the intervention was conducted with 10 patients to identify “hands-on” challenges requiring adaptation; a focus group was then held with 4 of those patients to further explore these challenges and solutions

All three focus group discussions were audio recorded and professionally transcribed and lasted approximately two hours each. Structural coding was used to mark responses to topical questions in the interview guide (Additional file 1: Appendix 1) [30]. Following a review of the a priori topics, the facilitator developed a codebook to categorize the data and identify salient themes and relationships [31, 32]. The main themes that emerged from the text identified specific recommendations for intervention adaptations. Both CAB members and patient stakeholders were provided a $50 incentive for participation, and each focus group was conducted in a private conference room.

### TM intervention description

TM is an interactive video monitoring system, connecting from the patient’s home via wireless transmission to the provider station (American TeleCare® LifeView Station monitoring provided in both English and Spanish). TM has two components: 1) a daily vital signs monitoring component (client-side operation), wherein patients monitor standard key indicators of possible condition exacerbation which are automatically transmitted to the server, and 2) a weekly telemonitoring face-to-face video visit, wherein patients attend a regularly scheduled televisit (real time) with the clinician.

### TM intervention component 1: daily vital signs

**Fig. 1:**
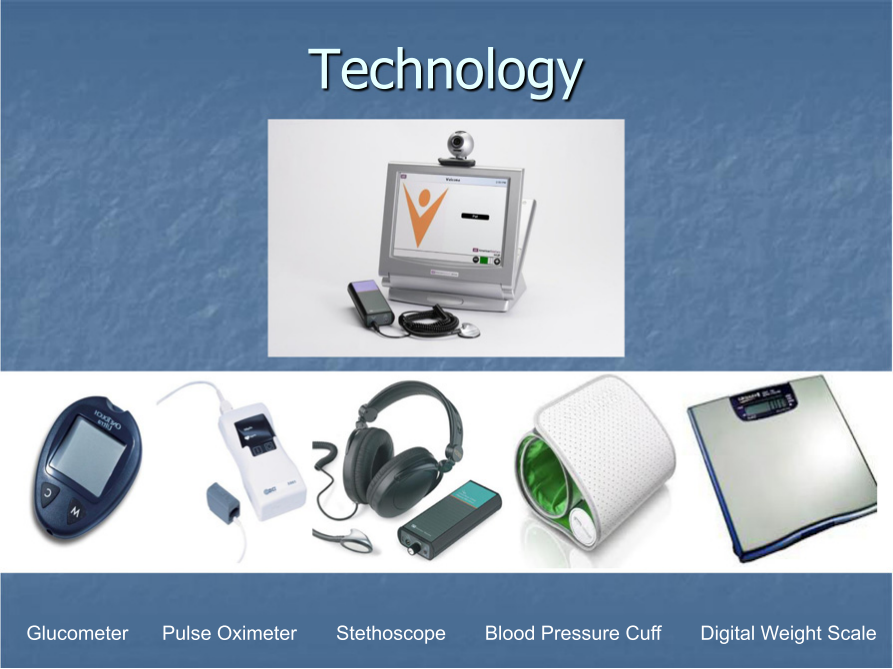
Telemonitoring intervention equipment

Patients are trained by the clinician and installer (in their homes) to utilize the equipment (Fig. 1), uploading key indicators of blood pressure, oxygen saturation rate, weight, and pulse/heart rate). These daily transmissions take about 10 min, and are stored on a secure, encrypted database.

### TM Intervention component 2: telemonitoring visit

Once a week, patients are asked to connect to the patient station for a weekly scheduled telemonitoring (TM) visit. The televisit allows the patient and the telemonitoring nurse to view one other and allows the clinician to listen to the patient’s heart and lung sounds through a stethoscope. During the televisit, the practitioner reviews the weekly uploads and the patient and provider discuss their behavior and vital signs.

### Pre-study usability (stakeholder perspective, Focus Groups 1 and 2)

The first CAB focus group (*n* = 14) was conducted during the formative phase of the project. A general discussion of community needs was followed by a dialogue regarding specific adaptation needs regarding both TM equipment and study design. CAB members were presented with the intervention (in English and Spanish), and a telemonitoring nurse, remotely connected, demonstrated core components and key adjustable characteristics of the initial intervention. A qualitative consultant led the focus group discussions, with content guided by predetermined topics outlined in an interview guide, including instructions to prioritize patient stakeholder contributions above medical and professional stakeholders [33].

A second CAB focus group, conducted a month later (immediately after adaptation but prior to intervention implementation), and led by the same qualitative researcher, involved a discussion framed by an interview guide as well as theater testing of the TM equipment with the CAB patient stakeholders. A draft of the adapted intervention was discussed to ensure that all of CAB feedback was effectively incorporated.

### Patient usability (patient perspective, Focus Group 3)

Focus group participation was offered to the initial 10 patient participants randomized to the TM arm of the study (4 agreed to participate) to obtain direct user feedback regarding implementation/usability barriers. Discussion topics included: 1) ease of intervention use (e.g., uploading, televisits); 2) intervention usefulness; 3) barriers to intervention implementation; and 4) adjustment recommendations. Patient participants in focus group 3 were recently hospitalized for HF. As with the stakeholder perspective, the focus group discussion was led by the qualitative research consultant, with discussion guided by predetermined topics outlined in an interview guide.

## Results

Results described below conform to the general principles of the ADAPT-ITT model (Table 1). The sections below describe the general themes and subthemes that arose from: 1) Community Advisory Board stakeholder focus groups; 2) Patient participant focus group.

## Focus groups with community advisory board

### Community needs assessment

The focus group discussion began with a general discussion of the needs of Black and Hispanic HF disparity patients; multiple challenges were identified, including repeated hospitalization, medication management, and co-morbidities. One issue that quickly emerged was that, in Black and Hispanic communities, HF is not necessarily a disease of older adults. One patient stakeholder in his late forties reported that years ago he experienced repeated hospitalizations, surgical procedures, and heavy treatment regimens before he achieved success in the management of his disease.

One clinician stakeholder noted that patients from disparity communities often have more co-morbid health conditions that complicate HF treatment regimens and patient adherence. Access to health care providers, including issues around scheduling and transportation, were cited as specific structural barriers that make it difficult to access HF care and treatment.

### Theater testing of the intervention (theoretical, with CAB patient stakeholders)

A one-on-one demonstration of the Telemonitoring equipment with the patient stakeholder CAB members (*n* = 4) was conducted by the community-based Telemonitoring nurse, study research analyst, and telehealth installation and patient orientation specialist. While each patient stakeholder was given a personal demonstration of the equipment, the facilitator recorded responses and took notes to assess first impressions and identify additional barriers to the equipment’s use. The equipment demonstration illustrated that some patient stakeholders initially are overwhelmed by the equipment, but the hands-on aspect of the demonstration helped them feel more confident.

For a few seconds there, it was overwhelming because you’re looking at everything in one shot… the scale…the pressure, and the other thing for your finger (pulse oximeter)… Then after I used it and looked at it again the second time … it felt better. I felt I get better each time using it…the confusion wasn’t there…You feel confident as you use it…

After a personal demonstration on how to use the equipment, an initially skeptical CAB member felt more confident that anyone (regardless of their current health status) could use it because they could always get help from the telemonitoring nurse.

### General assessment

Following a demonstration of the Telemonitoring equipment, the patient stakeholders in attendance provided initial assessments of the equipment. One Hispanic male patient stakeholder was very enthusiastic about using the equipment to save him time (and presumably cost) for going to see the doctor.

I like it….Yeah, it save me time…we don’t have to come here to the hospital, we can [stay at] the homes, talk to the doctor about what’s going on. They teach me [about] my pressure, about my heart, everything…

One Spanish-speaking patient stakeholder described his sentiments more fully in Spanish, which were translated by another CAB member to the rest of the group.

… *mararvilia* … It’s not like [he has] to go to the hospital or call the doctor or try to make an appointment or whatever. He’s a phone call away, a direct communication with a health practitioner…

#### Equipment changes

##### Font size

One suggestion made by a patient stakeholder focused on font size as it appears on the screen due to diabetes affecting her vision and ability to read.

Like I say all the time my problem is the visual… please put it [the font size of the numbers] bigger than this because even with glasses I have a big problem [seeing the numbers].

##### Spanish translation

The accuracy of the Spanish translation of the program materials (especially the screen instructions) was an important area of concern. For example, the term “el mangito” (“small mango” in most dialects) was used to describe the blood pressure cuff, when a more clear reference to the cuff could have been used.

##### Scale and voice

Two other suggestions were made by CAB members. The first involved the size of the weight scale. One patient stakeholder suggested that the scale was a little small, and it would be difficult to stabilize herself while standing on the scale. The second suggestion involved slowing down the speed of the oral instructions and reducing medical jargon of the telemonitoring unit during patient instruction.

##### Equipment issue resolution

The issues mentioned above were addressed by the study team in a series of conversations with the equipment manufacturers. Specifically, font sizes were increased, and medical jargon was simplified to accommodate a patient with a third grade reading level. Finally, an accurate Spanish translation of each screen was provided to the equipment manufacturer, who agreed to alter the screens as per the CAB’s suggestions. The one issue that could not be addressed in this way was the size of the scales; however, a suggestion by the installer was used as a potential solution. The installer suggested that patients place the scale in the corner of a room so that the patient could hold onto the walls for support and stabilization.

#### Suggested changes to the study structure

##### Have patient meet telemonitoring nurse in person first

It was suggested that participants be introduced to the study nurse early in the hospital stay, and be left with study materials to give them time to consider participation. This was suggested in order to build study rapport and trust, as well as confidence in using the equipment by creating a link to a “familiar face.”

##### Ensure continued access to doctor and/or clinic in addition to telemonitoring nurse, especially in case of an emergency

The CAB recommended that the study nurse strongly reiterate to the patient that participation in the study does not *replace* his/her regular provider, in the primary care or HF clinic setting.

##### Reassurances to undocumented patients

The CAB recommended that, before enrollment and during the introduction of the study, the study nurse reiterate to all patients that their immigration status is not recorded and will in no way be communicated to any third party during the study. This was integrated into the enrollment process by the study nurse.

##### Have a family member, friend or volunteer available who can assist the patient with using the equipment

A physician CAB member suggested that some patients would fare better if their televisits could be scheduled when caregivers (family or professional) were available to assist, particularly with regard to heart and lung sounds.

##### Addition of a pharmacist to the advisory board

One recommendation that emerged during the first focus group was the need to include the perspective of a pharmacist in the next discussion. The second focus group discussion did include a pharmacist who recommended that the study team should distribute pillboxes to help facilitate medication adherence.

##### Acknowledge perception that the technology used can be intimidating; increase the time that the patient is trained on the machine

Some patient stakeholders stated that they needed extra support; this lack of confidence in using the telemonitoring equipment can be addressed by providing more face-to-face demonstrations so that they can practice.

##### Study structure issue resolution

The issues mentioned above were largely addressed by the study team, such as 1) additional training time for participants, 2) addition of a pharmacist to the CAB, and 3) asking the nurse to carefully explain to every patient at enrollment that participation in the study would not *replace* his/her regular provider. In addition, the study nurse was instructed to approach the potential participant early in the hospital stay, leave materials about the study, and return to discuss enrollment, thereby leaving the potential participant with additional time to consider participation. Further, the study nurse took time to reiterate to all patients that immigration status was in no way a barrier to participation and is not recorded at all. This was beneficial in recruiting patients who may not have otherwise been willing to participate due to fears regarding their immigration status. One recommendation, scheduling at times when caregivers (family or professional) were available to assist, was able to be partially addressed: the TM nurse was able to schedule visits conductive to caregiver attendance, but only within the 9 am to 5 pm week day.

## Focus group: pilot study participants

To obtain direct user feedback regarding barriers to implementation or usability, we also invited the first ten patients randomized to TM to attend a focus group to further adapt the intervention. Four patients were able to attend. While the first two CAB focus groups allowed for the identification of issues that may be anticipated by stakeholders, this patient focus group allowed the study team to obtain important information regarding usability of the intervention in “real time” with live patients.

### General assessment

Consistent with theater testing findings, initial concerns with using the equipment dissipated with use, generally over a couple of days. Previously, focus group concerns about literacy were a potential barrier. However, the combined use of audio and text in the Telemonitoring program did help pilot participants understand the instructions.

The machine actually told you what to do. The machine itself is teaching you every single day. It says, you know, put the cuff on, sit quiet, elevate your arm…

#### Issues regarding equipment use

##### Warming hands before oximeter use

One patient stakeholder reported that, in her experience as a nurse, she had learned that inaccurate pulse oximeter readings could result if a patient’s hands were cold, and that patients should be instructed to warm up their hands before checking the oxygen saturation level for an accurate reading.

##### Using the stethoscope on the back

Patient stakeholders reported having some difficulty in handling the stethoscope when assessing lung sounds from the back. Patient stakeholders said that additional support (e.g., family member) was required to adequately reach the back.

##### Difficulty reading the scale

In response to a question from a fellow patient stakeholder about using the scale, one patient noted that the scale worked more consistently when you waited a few seconds after turning it on to finish calibration before stepping on.

##### Time barriers

Some pilot participants expressed that, once they felt comfortable with the equipment, it was easy to make time to upload. However, one participant indicated that it was difficult to make time every single day to upload.

Well I did skip a few days on doing it, like on Sunday I go to- I am in service so I didn’t do it on Sunday…

##### Space constraints

One participant expressed that space in his home was a barrier to usage so he actually had the equipment installed in a friend’s home and went over as often as possible to upload information.

Mine was in somebody else’s house, and they have six kids.

Others indicated that they were able to find space, but that some other family members were trying to use the equipment.

I have a 22 year old that is concerned about her weight so she was very interested in using it (laughter) …But it did (laughter) pique her interest very much.

##### Connectivity difficulties

All participants indicated that there were sporadic issues regarding connectivity; one participant indicated that the issue may have been less about connectivity and more about her own ability to navigate weekly calls with the nurse while using the equipment.

#### Issues regarding study structure

##### Length of intervention

Only one issue arose with regard to study structure in the pilot group. This participant felt that the time period (3 months) for observation of the intervention group was not long enough. With regard to the time period (3 months) of observation, the study team could not extend the time because of the nature of the grant funding; however, strong recommendations to future investigators to extend HF telemonitoring are in progress.

## Discussion

Although previous studies have documented the clinical efficacy of remote monitoring of HF [11], and discussed the importance of adapting interventions to facilitate cultural relevance [20, 21], this is the first study to describe the formative process of a community-based participatory research study aimed at optimizing telehealth utilization among African-American and Latino patients from disparity communities. Two major themes emerged from qualitative analyses of the focus group data. The first theme that arose involved suggested changes to the equipment that would maximize usability. Subthemes identified included issues that reflect the patient populations targeted, such as Spanish translation, font size (many of these patients are older diabetics with sight issues) and medical jargon, which is often unfamiliar to patients without medical backgrounds, and can be especially daunting to those patients with lower health literacy and for whom English is a second language.

The second theme that arose involved suggested changes to the RCT study structure in order to maximize participant engagement. Subthemes also identified issues that reflect concerns of the targeted patient populations. For example, the provision of reassurances regarding identity protection to undocumented patients is of particular importance in providing an intervention that involves the use of a camera. Similarly, assuring the patient that their involvement in telehealth monitoring would not replace their clinic care, which for many disparity patients is their only connection to medical care.

Study limitations included a relatively small sampling (40 %) of pilot patient participation in the focus group. Based on anectodal evidence, we believe that while this 40 % accurately represented the concerns of the current patient population, it may not represent concerns of patients in other settings. While the majority of suggested changes to both study structure and equipment were implemented, although there were a few suggestions that could not be achieved, due to resource limitations.

Before this study, no models existed to guide adaptation of a HF telemonitoring intervention in disparity communities. To formalize the adaptation process, the research team searched for an existing framework that utilizes an iterative and experiential process with stakeholders (Table 1) to facilitate relevance, sustainability, and acceptability for Black and Hispanic HF disparity patients.

THE ADAPT-ITT model was identified as a pragmatic framework utilizing an iterative and experiential process comprised of eight sequential phases to adapt HIV-related evidence-based interventions. The majority (7/8) of phases were applicable to the HF adaptation process.

**Phase I (Assessment)** utilizes focus groups consisting of key stakeholders to assist in formative evaluation in the form of a Community Advisory Board (CAB). Several researchers have documented the importance of assessment as part of the adaptation process [34–36]. Our experience conducting focus groups with key stakeholders, including the targeted patient population, resulted in a comprehensive assessment of the needs of the particular population as well as specific adaptation recommendations.

**Phase 2 (Decision)** involves the review, selection and decision to adopt or adapt an intervention. Since the basic interventions (key elements) were pre-determined by grant funding from the Patient Centered Outcomes Research Institute (PCORI), this was the only phase that could not be completed in its entirety (the decision to adapt many aspects of the intervention was made, but basic intervention selection was determined a priori).

**Phase 3 (Adaptation)** utilizes theater testing, a frequently used methodology in product testing, to adapt the intervention [37]. In the current study, CAB patient stakeholders attended a telemonitoring demonstration and subsequently “used” and “reacted to” the equipment and contribute ideas regarding intervention adaptation.

**Phase 4 (Production)** results in the production of a first draft of the adapted intervention. Although core elements of an intervention are based on the behavioral theory which is the basis of the intervention and thus cannot be changed, key characteristics of an intervention can be adapted. In our study, adaptation recommendations centered around two main themes: suggested changes to study structure and issues regarding equipment use.

**Phase 5 (Identification of Topical Experts)** is a process by which content area experts are identified to serve as consultants to the process. In the current study, the research team identified key stakeholders at project inception; in addition, the CAB identified that a pharmacist was needed to address issues related to medication management, so a pharmacist was added to the CAB.

**Phase 6 (Integration)** involves the creation of a more “finely tuned” draft of the intervention based on input from topical experts. In the current study, an updated draft was presented to the CAB during a second focus group to ensure that the adaptations recommended were properly implemented. For example, the telemonitoring screens containing the new Spanish translation were presented to the CAB for further feedback.

**Phase 7 (Training)** involves the training of staff to implement the updated version of the intervention. Specifically, the study nurse and installation expert were trained in the areas of changes to study structure and equipment.

**Phase 8 (Testing)** involves pilot testing the most current version of the intervention with “live patients”. A focus group was held with the initial intervention participants to identify additional barriers or challenges to implementation in the actual home setting. Following a prescribed adaptation framework allowed the study team to optimize the intervention so that it was relevant and acceptable to the targeted populations. The process allowed the study team to gain invaluable insights that would not have been otherwise evident. From the expertise of the patient stakeholders, to the practical understanding of the use of this technology in the home setting provided by the installation expert, the research team was able to adapt the intervention in such a way that it was deemed as truly usable by patient participants. The CBPR adaptation process also increased cultural sensitivity among staff ultimately resulting in greater assurances to patients with regard to complex issues such as immigration status, thereby increasing study participation rates.

## Conclusions

Addressing the two major themes (changes to the equipment to maximize usability, and changes to RCT study structure) and subthemes that emerged from our formative study is integral in order to maximize the efficacy of telehealth monitoring in Latino and African-American disparity populations. Further, this study exemplified the applicability of Wingood and DiClemente’s “ADAPT-ITT” framework in adapting a TM intervention for HF patients from African-American and Hispanic disparity communities. These findings demonstrate that a remote monitoring program, with live clinician support to facilitate patient engagement, can be adapted to reach patients who are most likely to experience access issues, with the ultimate goal of keeping patients healthy at home.

## Abbreviations

CAB, Community Advisory Board; CBPR, Community Based Participatory Research; HF, Heart failure; TM, telemonitoring

## Additional file

Additional file 1: Appendix 1. Telehealth Community Advisory Board (CAB) Focus Group Discussion Guide. **Appendix 2.** Telehealth Patient Focus Group Discussion Guide. (PDF 225 kb)
