# Supplementary material for: A qualitative analysis to optimize a telemonitoring intervention for heart failure patients from disparity communities
Source: BMC Med Inform Decis Mak. 2016 Jun 24;16:75. doi: 10.1186/s12911-016-0300-9 (PMC4919886; doi:10.1186/s12911-016-0300-9)
Supplement: Additional file 1: Appendix 1. — Telehealth Community Advisory Board (CAB) Focus Group Discussion Guide. Appendix 2. Telehealth Patient Focus Group Discussion Guide. (PDF 225 kb) [file 12911_2016_300_MOESM1_ESM.pdf]

APPENDIX 1
Telehealth Community Advisory Board (CAB) Focus Group Discussion Guide

Pre-Discussion Orientation

NOTE: Facilitators introduce themselves and describe what a focus group discussion is and how it works. Tell respondents that the focus group discussion will last approximately two hours. Acknowledge the discussion will be audiotape-recorded and provide assurances that all information that they share with us will be kept private. Ensure that all participants understand and have provided consent to the audio recording of the discussion.

Remind the respondents that at the end of the discussion, they will be asked to give feedback about any of the questions they consider difficult or unclear. They will also be asked about whether there is any unclear language in the description of the device or the training instructions and if they have suggestions for other language that would be more understandable.

I. Self-Introductions and Warm up

1. What is a focus group discussion?
2. How do focus group discussions work?
    - interested in your viewpoint, you represent other patients with chronic heart failure who may have views just like you
    - research project, not selling anything, just want your perceptions
    - no right or wrong answers
    - honest answers
    - If a particular question or questions make you uncomfortable, you don't have to provide an answer – just ask me to move on to the next question.
3. Group defines/outlines rules for mutual respect
    - One person talk at a time
    - Put cell phones on vibrate
    - No judgment
4. Microphones, recording, assurance of privacy

INTRODUCTION (NOTE: Text written using lay language)

ICE BREAKER – self introductions

Moderator: Chronic heart failure is common among hospitalized patients over 65, and is a leading cause of disability and death. Once discharged from the hospital, it is important for patients to continue to monitor their health progress. Unfortunately, most patients do not have the knowledge or the skills to manage their disease at home. We are conducting a study at North Shore LIJ in collaboration with Nassau University Medical Center to see if we can use Telehealth, which is a computer-based health technology to help patients in the community monitor changes in their weight, heart rate, lung sounds, and blood pressure so that providers can adjust treatments and keep people out of the hospital. Without leaving the homes, patients can upload their vital signs and be monitored by clinicians through telehealth visits using voice and video equipment combined with other medical technology. Telehealth has been shown to help other patients with chronic heart failure. Today, we are going to discuss your thoughts and ideas about the use of Telehealth to help patients monitor their health from home.

There are **<u>three</u>** ways that you can contribute to today's discussion:

1. You can share your personal experience (again, we remind you that what is said here today will remain confidential and anything you tell me won't be linked back to you).
2. You can also share your thoughts and ideas as they pertain to other patients with chronic heart failure. For instance, you can talk about someone that you know or that you have heard about (use only pseudonyms).
3. You can share your thoughts about any adjustments that need to be made to the telehealth program so that it is most relevant and appropriate for use by the target patient population.

These comments will help us to understand how to better help patients with chronic heart failure and reduce the risk of future hospitalizations.

Remember today that you are the expert and were the students!

<u>Do you have any questions about the study or the interview before we started?</u>

<u>May we turn on the tape recorder now?</u>

<table>
<tr><td colspan="3"></td></tr>
<tr><td colspan="3">Background experience, description of the patient population and research participation</td></tr>
<tr><th>#</th><th>Questions</th><th>Probes</th></tr>
<tr><td>1</td><td>First, let’s take a moment to talk about our own experiences with chronic heart failure, as patients, providers or health advocates from the community.</td><td>• Number of years

Patients
• Hospitalization
  ◦ Recent?
  ◦ How many times?
  ◦ Number of ER visits?
• Diagnosis
• Treatment
• Current health status in relation to CHF

Providers
• Training/work experience

Community advocates</td></tr>
<tr><td>2</td><td>How would you describe most patients with chronic heart failure in your community?</td><td>• Gender
• Race/Ethnicity
• Age
• Marital status
• Employment status
• Education
• Household composition
• Social support
• Insurance

<u>Clinical issues: </u>Co morbiditiies
• Hypertension
• Diabetes
• Asthma</td></tr>
</table>

		• Sleep apnea<br>• Depression or other mental health issue<br>• Chronic inflammation<br>• Fractures<br>• Hep B or C<br>• HIV infection<br><br><u>Life-style</u><br>• Alcohol and drug use<br>• Smoking<br><br><u>Care & Treatment</u><br>• Where do they go for care and treatment?<br>• General provider vs. specialist<br>• How often do you for care (e.g., every 3 months)<br>• Multiple sites<br>• Recent hospitalizations<br>• ER visits<br><br><u>Barriers to treatment</u><br>• Transportation (distance or difficult to access)<br>• Money (cost of getting to visits; have to work)<br>• Caregiver responsibilities (for children, parents or sick partner)<br>• Concerns about privacy or disclosure
3	What do you think are the most important treatment needs for patients with chronic heart failure in this community?	• Access Issues<br>• Provider Issues<br>• Reduction of hospitalizations<br>• Self-care; increasing self-efficacy<br>• Quality of life
4	What do you think are barriers to participation in research in this patient population?	• Mistrust<br>• Breach of confidentiality

<table>
<tr><td>5</td><td>What suggestions would you make about reducing any barriers to participation?</td><td>• Trust building</td></tr>
<tr><td colspan="3">Demonstration of the Telehealth product (25 mins)

Now we are going to take some time to demonstrate how to use the Telehealth device as if you were a potential patient. We will have time at the end to answer any questions you may have. We will also ask you questions about how we can improve our training methods.

NOTE: Provide as much time as is needed to address any questions. When appropriate, move into the next section.</td></tr>
<tr><td colspan="3">Assessment of Telehealth device and training

Thank you for your questions.

Now we would like to ask some questions about the Telehealth device so that we improve our methods of training or address potential barriers to its use. These questions will help us identify how we might need to modify the Telehealth program so that it is appropriate for the patient with chronic heart failure in this community.</td></tr>
<tr><td>6</td><td>First, let us ask: What are your initial impressions of the device?</td><td>• Positive or negative impressions

<u>Follow up question:</u>
• Is there anything about telehealth visits that makes you uncomfortable?</td></tr>
<tr><td>7</td><td>After this demonstration, do you think that you would be able to use the Telehealth device?</td><td>Probe:
• Positive or negative impressions
• Barriers
• What could make it challenging for patients to use the Telehealth device?

Do you think that other patients with chronic heart failure in this community will be able to use?
• Positive or negative impressions
• Barriers
• What could make it challenging for</td></tr>
</table>

		patients to use the Telehealth device?
8	How easy do you think it will be to learn to use this equipment?	• Positive or negative impressions
9	Were the instructions clear?	• Points of confusion or clarity<br>• Was there too much jargon?<br>• Were the Spanish translations accurate?
10	Was the demonstration helpful?	• Points of confusion or clarity
11	How do you think the telehealth system can affect health and wellbeing for patients with CHF?	• Positive or negative impressions<br>• Empowerment<br>• Improve self efficacy<br>• Create feelings of alienation<br>• Create feelings of suspicions<br><br><u>Follow up questions</u><br><br>1. How can the telehealth program affect patients' ability to manage their health?<br>2. How can the self-monitoring of your health affect the health decisions or behaviors of patients with CHF?
12	How easy do you think it will be for patients to use the telehealth equipment on a daily basis?	• What environmental or social variables could make it difficult for patients to use the machine on a daily basis?
13	What impact, if any, do you think the nurse's visits will have on their health decisions or behaviors?	• Positive or negative impressions
14	Does the equipment seem easy to use?	• Is the screen large enough?<br>• Is the font large enough?<br>• How easy did it seem to take one's own blood pressure, listen to heart, weigh oneself?<br>• How easy did it seem to be able to upload the measurements?

15	How easy did it seem to talk to the nurse during a video visit? Feelings about having someone see you in your home?	• Positive or negative impressions
16	Do you think that patients will feel that the experience of talking with the nurse during the telehealth visit will be as satisfying as talking to them in person?	• Why?
17	Do you feel that patients will be concerned about their privacy when talking with the nurse during the telehealth visit?	• Why?<br>• If yes, suggestions?
**Technological concerns**<br><br>No we would like to ask you specific questions about potential problems that the patients might experience with the technology used in the Telehealth program		
18	After today's presentation, do you think that the technology in Telehealth is "user-friendly" for patients in our community?	• Do you think that individuals intimidated by computers will be reluctant to use this device?<br>• Do you think that many will think this is too "high-tech" for them?<br>• Do you think that it might seem too complicated or confusing?<br>• How many patients do you think are computer or technology savvy?
19	Do you think patients in this study will have any problems with the following:<br>• Size of the screen<br>• Touch-screen technology<br>• Size of the font<br>• Location of the buttons<br>• Using any of the medical equipment<br>• Reading the results from any of the equipment<br>• Uploading the information	• Probe for specific problems and ideas for how to address or fix these potential issues<br>• Probe for attention to special needs (e.g., visual or hearing disability, extreme arthritis, limited mobility, etc)

20	How many patients with CHF in our community have phone lines (or POTS) in the home?	• What do most people have?<br>• Land lines versus cell phones?
21	How many patients with CHF in our community have internet connection in the home?	• For those patients with Internet access, do you think that most use it for cable television or for online computer use?
22	Do you think that patients will be able to use air cards instead?	•
**Language Issues**<br><br>Now we would like to ask you some questions about the language used in the training and on the Telehealth program.		
23	Was the language used clear and concise?	• Was there too much medical jargon?
24	How could we improve how we describe the product or talk about Telehealth in general?	•
25	We have also translated all of the materials into Spanish. For those of you who are Spanish-speakers, could you help us determine if the Spanish translations are accurate?	• Are the translated screens in Spanish in right dialect for our Spanish-speaking population?<br>• Are the Spanish translated screens similar or different from the English version?<br>• How important will it be for the patient population to also have the nurse speak a Spanish dialect that matches the patient population? How difficult is it to understand different dialects?<br>• Which national dialects are most common among our patient populations?<br>◦ Puerto Rico<br>◦ Dominican Republic<br>◦ Mexico<br>◦ South America (Venezuela, Columbia, Equator, Argentina, etc)<br>◦ Central America (Honduras, Costa Rica, El Salvador, etc)

**Potential environmental issues**<br><br>Now that you have had an opportunity to see how the device is used we want to ask you about some of the environmental issues in the home that may or may not make it difficult for patients to use the Telehealth program. These questions pertain to space constraints, time limitations and social support.		
26	The first question is about space. Do you think that having enough space in the home will be an issue or not for the proposed patient in this study?	• Does the equipment take up too much room?<br>• Will it be difficult for patients to keep their equipment safe?
27	Another possible barrier to using the Telehealth program is not having enough time to upload the information. We anticipate that some patients will have busy lives and may not have enough time. Do you think that patients may have problems making a time commitment for the Telehealth visits?	• How can we help busy patients fit the Telehealth program into their schedule?<br>• Caregiver schedules<br>• Patient schedules<br>• Are the visits scheduled at a convenient time
28	We know that having social support can be essential to medication and treatment adherence. Do you think that having someone in the home other than patient is necessary to help them use the Telehealth program?	• What kind of social support is ideal?<br>• What should be done to help patients that do not have social support?<br>• Finally, many patients may have health aides to help them in the home. Many of these health aides work with patients for short periods of time.<br>• How can we help patients educate their new health aides on the Telehealth program so that they can continue to get help when they need it?
29	Did you have any comments or suggestions for how to help patients with chronic heart failure to use the Telehealth program?	• Training<br>• Language used<br>• Help with use of the device

	**Final comments or suggestions**<br><br>We’ve reached the end of the discussion. Do you have anything else that you would like to add about the topics that we have discussed here today?<br><br>**THANK YOU FOR YOUR TIME AND FOR YOUR IMPORTANT INPUT!**	

APPENDIX 2
Telehealth Patient Focus Group Discussion Guide

Pre-Discussion Orientation

NOTE: Facilitators introduce themselves and describe what a focus group discussion is and how it works. Tell respondents that the focus group discussion will last approximately two hours. Acknowledge the discussion will be audiotape-recorded and provide assurances that all information that they share with us will be kept private. Ensure that all participants understand and have provided consent to the audio recording of the discussion.

Remind the respondents that at the end of the discussion, they will be asked to give feedback about any of the questions they consider difficult or unclear. They will also be asked about whether there is any unclear language in the description of the device or the training instructions and if they have suggestions for other language that would be more understandable.

I. Self-Introductions and Warm up

1. What is a focus group discussion?
2. How do focus group discussions work?
    - interested in your viewpoint, you represent other patients with chronic heart failure who may have views just like you
    - research project, not selling anything, just want your perceptions
    - no right or wrong answers
    - honest answers
    - If a particular question or questions make you uncomfortable, you don't have to provide an answer – just ask me to move on to the next question.
3. Group defines/outlines rules for mutual respect
    - One person talk at a time
    - Put cell phones on vibrate
    - No judgment
4. Microphones, recording, assurance of privacy

INTRODUCTION (NOTE: Text written using lay language)

ICE BREAKER – self introductions

Moderator: Chronic heart failure is common among hospitalized patients over 65, and is a leading cause of disability and death. Once discharged from the hospital, it is important for patients to continue to monitor their health progress. Unfortunately, most patients do not have the knowledge or the skills to manage their disease at home. We are conducting at study at North Shore-LIJ in collaboration with Nassau University Medical Center to see if we can use Telehealth, which is a computer-based health technology to help patients in the community monitor changes in their weight, heart rate, lung sounds, and blood pressure so that providers can adjust treatments and keep people out of the hospital. Without leaving the homes, patients can upload their vital signs and be monitored by clinicians through telehealth visits using voice and video equipment combined with other medical technology. Telehealth has been shown to help other patients with chronic heart failure. Today, we are going to discuss your thoughts and ideas about the use of Telehealth to help patients monitor their health from home and to hear about your experience, thus far, with the telehealth intervention.

There are **two** ways that you can contribute to today's discussion:

1. You can share your personal experience (again, we remind you that what is said here today will remain confidential and anything you tell me won't be linked back to you).
2. You can also share your thoughts and ideas as they pertain to other patients with chronic heart failure and what their experiences with telehealth might be.

Both comments will help us to understand how to better help patients with chronic heart failure and reduce the risk of future hospitalizations.

Remember today that you are the expert and were the students!

Do you have any questions about the study or the interview before we started?

May we turn on the tape recorder now?

**Background experience and description of the patient population**		
#	Questions	Probes
1	First, let's take a moment to talk about our own experiences with chronic heart failure, as patients.	• Number of years<br><br>Patients<br>• Hospitalization<br>• Recent?<br>• How many times?<br>• Number of ER visits?<br>• Diagnosis<br>• Treatment<br>• Current health status in relation to CHF<br><br>Providers<br>• Training/work experience<br><br>Community advocates
2	Besides chronic heart failure, are you being treated for any other illnesses or conditions? What are these illnesses?	Chronic ailments<br>• Hypertension<br>• Diabetes<br>• Asthma<br>• Sleep apnea<br>• Depression or other mental health issue<br>• Chronic inflammation<br>• Fractures<br><br>Life-style<br>• Smoking<br>• Substance use
3	Now let's talk a little bit about your experiences with your care for chronic heart failure.	• Where<br>• When<br>• General provider vs. specialist

		• Multiple sites<br>• Recent hospitalizations<br>• ER visits<br>• General positive or negative experiences<br>•
4	When you receive care, do you usually see the same doctor(s) or nurse for your care? How many doctors do you see? Can you tell me a little about them?	Probe for:<br>• Age<br>• Gender<br>• Ethnicity<br><br>REPEAT if multiple providers – general provider versus cardiologist or other specialist<br>•
5	What is the quality of your relationship with your cardiovascular care provider?	• Is it Excellent, Good, Okay, Poor or horrible?<br>• Are they accessible?<br>• Can count on them when you need help?<br>• Do they take time to discuss issues with you?<br>• Do you get help promptly or do you have to wait?<br>• Do you feel like you can talk to them about anything?<br>• Can you give an example?<br>•
6	About how often do you go for care?	If they get stuck use time frames below<br>• Every month<br>• Twice a month<br>• Every 3 months<br>•
7	Do you get your care for these other illnesses or conditions at the same time or place as you get your cardiovascular care, or do you receive this care separately?	PROBE:<br>• Where?<br>• With whom?<br>• How long have you been there?<br>• How difficult is it to get to your doctor?

		About how often do you go for care?<br>• Every month<br>• Twice a month<br>• Every 3 months<br><br>• How did you incorporate the medical appointments into your routine?<br>•
8	How have you managed your care and treatment having both your chronic heart failure and other illnesses (as applicable)?	PROBE:<br><br>• Are you seeing the same provider for all of your health care needs or are you seeing several providers?<br>• Do your providers communicate with each other?<br>• Do you find it challenging or not working with more than one provider to take care of all of your health care needs?<br>•
9	What are some of the things that make it easier or difficult for you to go to your cardiovascular care visits?	• Transportation (distance or difficult to access)<br>• Money (cost of getting to visits; have to work)<br>• Caregiver responsibilities (for children, parents or sick partner)<br>• Concerns about privacy or disclosure<br>•
**Assessment of Telehealth device and training**<br><br>Now we would like to ask some questions about the Telehealth device so that we improve our methods of training or address potential barriers to its use. These questions will help us identify how we might need to modify the Telehealth program so that it is more helpful for you and other patients with chronic heart failure in this community.		
10	First, let us ask: What were your initial impressions of using the device?	• Positive or negative impressions<br><br>Follow up question:

		• Is there anything about telehealth visits that makes you uncomfortable?
11	What are some of the positive and negative aspects of using the device?	Probe:<br>• Positive or negative impressions<br>• Barriers<br>• What made it challenging for you to use the Telehealth device?<br><br>Do you think that other patients with chronic heart failure in this community will be able to use it?<br>• Positive or negative impressions<br>• Barriers<br>• What could make it challenging for patients to use the Telehealth device?
12	How easy was it to learn how to use the telehealth equipment?	• Positive or negative impressions
13	Were the instructions clear?	• Points of confusion or clarity<br>• Was there too much jargon?<br>• Were the Spanish translations accurate?
14	Was the demonstration helpful?	• Points of confusion or clarity<br>• Was the person who provided the training helpful?<br>• Were the able to answer all of your questions and concerns?
15	How do you think the telehealth system will affect your health and wellbeing?	• Positive or negative impressions<br>◦ Empowerment<br>◦ Improve self efficacy<br>◦ Create feelings of alienation<br>◦ Create feelings of suspicions<br><br><u>Follow up questions</u>

		1. How can the telehealth program affect your ability to manage your own health?<br>2. How can the self-monitoring of your health affect your health decisions or behaviors?
16	How easy or hard has it been for you to use the telehealth equipment on a daily basis?	• What things or people in your home made it difficult or easy for you to use the machine on a daily basis?<br><br>• How easy do you think it will be for patients to use the telehealth equipment on a daily basis?
17	What impact, if any, do you think the nurse's visits have had on your health decisions or behaviors?	• Positive or negative impressions<br>• What impact, if any, do you think the nurse's visits will have on their health decisions or behaviors?<br>•
18	Does the equipment seem easy to use?	• Is the screen large enough?<br>• Is the font large enough?<br>• How easy did it seem to take one's own blood pressure, listen to heart, weigh oneself?<br>• How easy did it seem to be able to upload the measurements?
19	How easy was it to talk to the nurse during a video visit?	• Positive or negative impressions
20	Was the experience of talking with the nurse during the telehealth visit as satisfying as talking to them in person?	• Why?
21	Were you ever concerned about their privacy when talking with the nurse during the telehealth visit?	•

**Technological concerns**<br><br>No we would like to ask you specific questions about any technological problems you may or may not have experienced with the Telehealth equipment.		
22	After several weeks of using the Telehealth program, do you think that the program is "user-friendly" or not for patients in our community?	• Were you ever intimidated by using the touch screen?<br>• Did you think that this program was too "high-tech" for you?<br>• At any point did you think that it was too complicated or confusing?<br><br>• Do you think that individuals intimidated by computers will be reluctant to use this device?<br>• Do you think that many will think this is too "high-tech" for them?<br>• Do you think that it might seem too complicated or confusing?<br>• How many patients do you think are computer or technology savvy?
23	Did you have any problems with the following:<br><br>• Size of the screen<br>• Touch-screen technology<br>• Size of the font<br>• Location of the buttons<br>• Using any of the medical equipment<br>• Reading the results from any of the equipment<br>• Uploading the information	• Probe for specific problems and ideas for how to address or fix these potential issues<br>• Probe for attention to special needs (e.g., visual or hearing disability, extreme arthritis, limited mobility, etc)
24	If you used a phone connection, did you ever experience a problem with the phone line?	• Probe for complication or ease with use
25	If you used the Internet, did you ever have a problem with the Internet	• Probe for complication or ease with use

	connection?	• Was this a provider issue or overuse (too many individuals using the same wifi) issue?<br>•
26	If you used an air card for connection, did you experience any problems?	• Probe for complication or ease with use
**Language Issues**<br><br>Now we would like to ask you some questions about the language used in the training and on the Telehealth program.		
27	Was the language used clear and concise?	• Was there too much medical jargon?<br>• Did you understand all of the instructions?
28	How could we improve how we describe the product or talk about Telehealth in general?	• Less jargon<br>• More graphics
29	[FOR SPANISH SPEAKERS ONLY]<br><br>How were the Spanish translations of the materials? Were they accurate or confusing?	• Are the Spanish translated screens similar or different from the English version?<br>• How important will it be for the patient population to also have the nurse speak a Spanish dialect that matches the patient population? How difficult is it to understand different dialects?<br>• Which national dialects are most common among our patient populations?<br>• Puerto Rico<br>• Dominican Republic<br>• Mexico<br>• South America (Venezuela, Columbia, Equator, Argentina, etc)<br>• Central America (Honduras, Costa Rica, El Salvador, etc)

**Potential environmental issues**<br><br>Now that you have had an opportunity to use the equipment we want to ask you about some of the environmental issues in the home that may or may not have made it difficult for you to use the Telehealth program. These questions pertain to space constraints, time limitations and social support.		
30	The first question is about space. Did you have enough space in your home for the equipment? Was the storage of the equipment an issue?	• Does the equipment take up too much room?<br>• Will it be difficult for patients to keep their equipment safe?
31	Some patients have very busy lives and many do not have enough time for their Telehealth visits. How easy or difficult was it to put aside time everyday to measure and upload your health information?	• How can we help busy patients fit the Telehealth program into their schedule?<br>• Caregiver schedules<br>• Patient schedules<br>• Are the visits scheduled at a convenient time
32	We know that having social support can be essential to medication and treatment adherence. Who do you have in your home or in your life in general who helps you with your health care? Specifically, who has helped or can help you with the Telehealth program?	• Probe for what kind of social support they have<br>• What do they do if they do not have help?<br><br><u>Questions about health aides:</u><br>• Do you have a health aide? Do they help you with the Telehealth visits?<br>• How do they help you?<br>• Were they trained?<br>• By whom?
33	From your experience using the Telehealth program, do you have any comments or suggestions for how to help patients with chronic heart failure to use the Telehealth program?	• Training<br>• Language used<br>• Help with use of the device
	**Final comments or suggestions**<br>We've reached the end of the discussion. Do you have anything else that you would like to add about the topics that we have discussed here today?<br>**Thank you for your time and important input!**	
